# Behavioural and temporal partitioning of dolphin social groups in the northern Adriatic Sea

**DOI:** 10.1007/s00227-018-3450-8

**Published:** 2018-12-18

**Authors:** Tilen Genov, Tina Centrih, Polona Kotnjek, Ana Hace

**Affiliations:** 1 Morigenos—Slovenian Marine Mammal Society, Piran, Slovenia; 2 Sea Mammal Research Unit, Scottish Oceans Institute, University of St Andrews, St Andrews, UK

## Abstract

Complex social structure is a prominent feature in several mammal species. Such structure may lead to behavioural diversity not only among populations, but also within a single population, where different subsets of a population may exhibit different types of behaviour. As a consequence, understanding social structure is not only interesting biologically, but may also help conservation and management efforts, because not all segments of a population necessarily respond to or interact with human activities in the same way, or at the same time. In this study, we examined the social structure of common bottlenose dolphins (*Tursiops truncatus*) in the Gulf of Trieste and adjacent waters (northern Adriatic Sea), based on a 9-year dataset, using social network metrics and association indices. We assessed whether different segments of the population show differences in behaviour and interactions with fisheries. Dolphin social network was structured into distinct social clusters of mixed sexes. We found no evidence of male alliances. The two largest social clusters overlapped spatially, but not temporally, as they used the same area at different times of day. Such diel temporal partitioning does not appear to have been documented in cetaceans previously. The two clusters also differed in ways they interact with fisheries, as one regularly interacted with trawlers, while the other did not. This study demonstrates how different segments of animal populations can interact differently with human activities and in turn respond differently to anthropogenic impacts.

## Introduction

Complex social structure is prominent in many mammals including primates (Chapman and Rothman 2009), elephants (Wittemyer et al. 2005), hyaenas (Smith et al. 2008), bats (Popa-Lisseanu et al. 2008) and cetaceans (Mann et al. 2000), and plays an important role in population dynamics and behavioural patterns. It governs the way the spread or containment of behaviours is facilitated, e.g. through social learning (Heyes 1994; Laland 2004). This may lead to behavioural diversity not only among, but within populations, where different population segments exhibit different behaviours (Mann and Sargeant 2003; Cantor and Whitehead 2013). Understanding this is not only interesting biologically, but may help conservation efforts (Whitehead 2010), because not all population segments necessarily respond to, or interact with, human activities the same way, or at the same time. There is concern over the effects of anthropogenic disturbance to populations, yet it is difficult to assess population-level impacts without understanding what proportion of animals may be affected.

Bottlenose dolphins (*Tursiops* sp.) are well-studied social mammals (Wells et al. 1987; Smolker et al. 1992; Lusseau et al. 2003; Connor et al. 2006; Lusseau 2006). Most information on their social structure comes from studies in Sarasota, Florida (Wells et al. 1987; Wells 2003) and Shark Bay, Australia (Connor et al. 1999; Mann et al. 2000), but many populations remain poorly studied. They are generally described as fission–fusion species, where group composition changes frequently (Connor et al. 2000), but we argue that their social structure varies considerably among populations. For example, dolphins in Florida appear to feature marked sex-age segregation, where males form paired alliances, females form bands and nursery groups and juveniles form smaller groups (Wells et al. 1987). In Shark Bay, males form hierarchical alliances (Connor et al. 1999, 2011; Randić et al. 2012). At the other end of the spectrum, dolphins in Doubtful Sound, New Zealand, form mixed-sex groups with strong associations not only within, but also between sexes (Lusseau et al. 2003). This shows that patterns cannot be generalised and that our understanding of bottlenose dolphin social structure remains incomplete.

Social network analysis allows groups of social animals to be studied as a network of nodes and ties (Wey et al. 2008; Krause et al. 2009a). When coupled with information on behaviour and interactions with human activities, it is a powerful tool in the study and conservation of social animals. Common bottlenose dolphins (*T. truncatus*) inhabit the Gulf of Trieste and adjacent areas of northern Adriatic Sea, where they have been studied since 2002 (Genov et al. 2008, 2017). Here, we examine the social structure of local dolphins and assess whether different population segments show differences in behaviour and interactions with human activities.

## Materials and methods

### Data collection

**Fig. 1:**
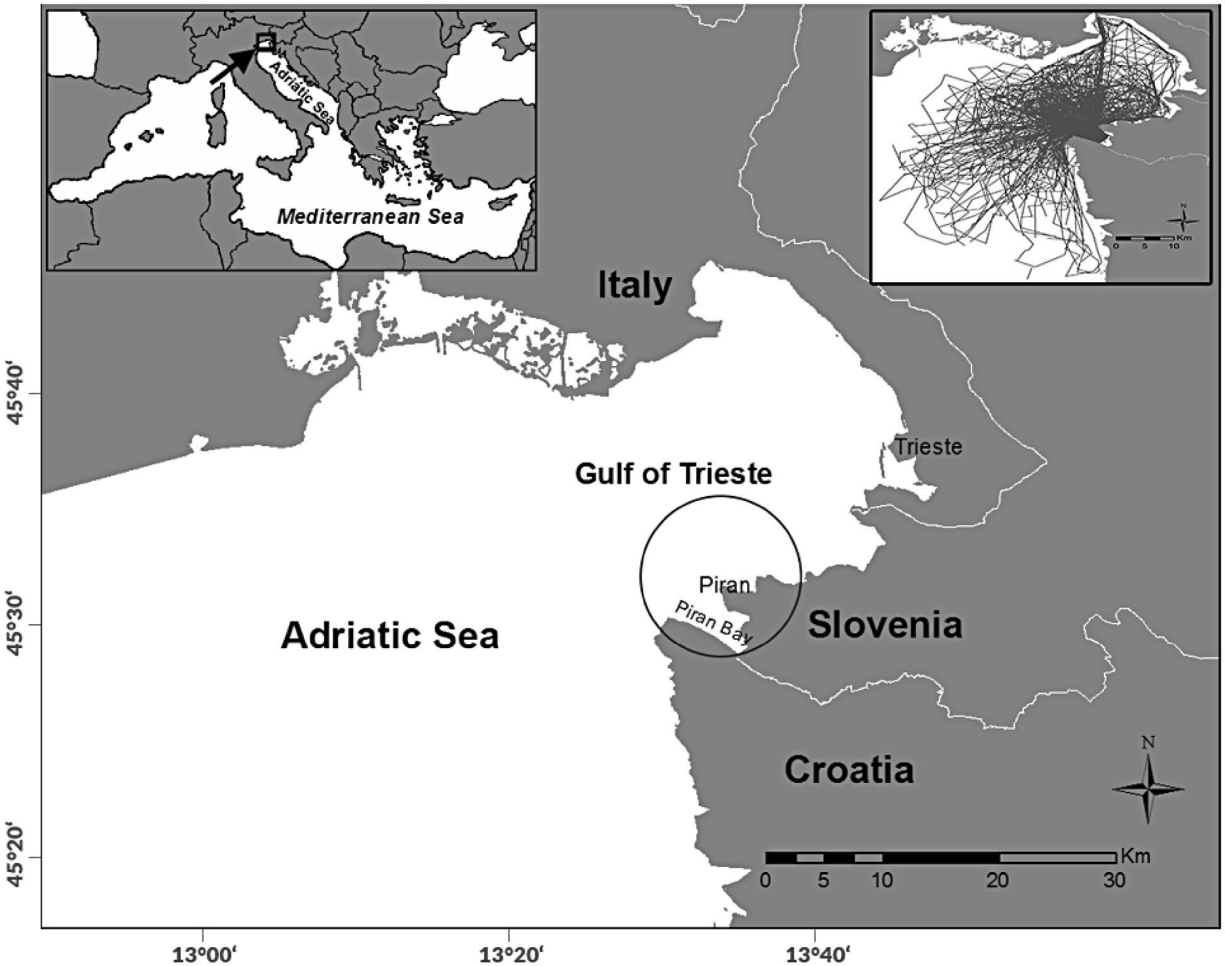
Study area in the northern Adriatic Sea, with locations cited in the text. The upper left inset shows the location of the study area in the Adriatic Sea. The upper right inset shows the spatial distribution of boat survey effort (navigation tracks). The circle depicts the ‘core study area’ where effort was most intense and included both boat-based and land-based surveys (see main text for details)

Data were collected between February 2003 and September 2011 in the Gulf of Trieste and adjacent waters, northern Adriatic Sea (Fig. 1). Based on mark–recapture abundance estimates, about 40–100 dolphins use this area annually, the majority carrying natural marks suitable for long-term identification (Genov et al. 2008; Genov 2011). The study area, survey methods and photo-identification procedures are described in detail in Genov et al. (2008). In short, boat surveys were complemented with land-based surveys to maximise the probability of encountering and photographically capturing dolphins. Survey coverage varied among years, due to weather, dolphin distribution and logistical constraints (Table 1). Each year we attempted to survey the entire area as homogeneously as possible. Surveys were done predominantly during summer (July–September), but periodically also in other months. Due to typical summer weather, surveys were commonly done in the morning and early afternoon, ceased in the early afternoon due to wind and resumed in late afternoon. Southern portion of the Gulf of Trieste, including waters along the Slovenian coast and Piran Bay, was surveyed consistently over the years and received more coverage than the outer edges of the study area (Fig. 1), due to the location of the home port and the land-based observation point. This sub-area, encompassing a roughly 5 km radius around the Piran peninsula, was regularly surveyed by both boat-based and land-based surveys and can be considered ‘core study area’ for the purposes of some of the results presented later on.

**Table 1:** Survey effort between 2003 and 2011, showing boat effort (in km surveyed) and land effort (in hours and minutes surveyed), number of group observed and number of individuals identified

Year	Survey effort		Nr. groups observed	Nr. individuals identified
	Boat [km (morning/afternoon)]	Land [hours + minutes (morning/afternoon)]		
2003	na	44 h 47 min (26 h 2 min/17 h 45 min)	4	8
2004	na	52 h 28 min (35 h 59 min/16 h 29 min)	5	13
2005	261 (174/87)	33 h 23 min (20 h 50 min/12 h 33 min)	15	21
2006	219 (153/66)	44 h 17 min (23 h 12 min/21 h 5 min)	14	20
2007	256 (170/86)	56 h 36 min (41 h 6 min/15 h 30 min)	7	21
2008	502 (306/196)	65 h 37 min (43 h 25 min/22 h 12 min)	18	32
2009	641 (408/233)	88 h 39 min (55 h 52 min/32 h 47 min)	14	31
2010	607 (358/249)	142 h 20 min (89 h 15 min/53 h 5 min)	27	19
2011	600 (361/239)	148 h 25 min (97 h 48 min/50 h 37 min)	11	22
Total	3086	675 h 32 min	115	38

Numbers in parentheses show the breakdown of survey effort into morning and afternoon, respectively. (Nr. groups observed and individuals identified only refers to the already restricted dataset of individuals included in social network analysis, not all identified dolphins—see “Materials and methods”)

Photographs of dorsal fins were obtained during focal follows and allowed individual identification (Würsig and Jefferson 1990). Members of a dolphin group were considered associated. Group was defined as dolphins observed behaving in a generally coordinated fashion (moving in the same direction or staying in the same area, usually engaged in the same general activity). In practice this meant that group members were always within about 100 m from the nearest other dolphin. Field observations and photo-identification showed that group composition rarely changed during several hours of observation (Genov et al. 2008).

Sex was determined by (a) observations of mother–calf pairs (adults consistently accompanied by calves were assumed to be mothers and, therefore, females); (b) photographs of the genital area or (c) molecular methods from biopsy sampling carried out opportunistically within Slovenian waters (permit 35601-102/2010-4 by the Slovenian Environmental Agency). Skin and blubber samples were obtained using a 68-kg draw weight crossbow, using custom-made bolts and stainless steel sampling tips with length of 25 mm and internal diameter of 7 mm. Tips were sterilised using 96% ethanol and burning prior to being used. Dolphins were sampled in the dorso-lateral area below the dorsal fin, at distances of 4–10 m. All biopsy attempts were accompanied by concurrent photo-identification. Sampling was only attempted on adults without accompanying offspring. Skin samples were removed and excised with sterilised forceps and surgical scissors, placed in 96% ethanol and stored at − 20 °C until analysis. Samples were analysed as described in Gaspari et al. (2015).

Numerous trawlers operated in the area year-round. They can be divided into (a) single bottom trawlers and (b) pelagic/mid-water pair trawlers. Bottom trawlers were typically 9–15 m long, operated alone and trawled nets on the seabed, targeting several demersal species. Pair trawlers were typically 30 m long, operated in pairs and trawled nets in mid-water. They mostly targeted European anchovies (*Engraulis encrassicolus*) and sardines (*Sardina pilchardus*). Dolphins interacted with both trawler types (Genov et al. 2008). Interaction was defined as dolphins following operating trawlers, approximately 200–400 m from stern (closer for bottom trawlers and further for pair trawlers, but the exact distance could vary), and typically alternating long dives (> 1 min) with sequences of short dives (5–30 s).

### Data restrictions

Only high-quality photographs (sharp image, fin perpendicular to the camera lens, entire fin visible and not obstructed by water spray or other animals, fin height < 7% of the frame height) were used. Association patterns were analysed for well-marked individuals only (123 individuals). However, as individuals with low encounter rates can introduce biases (Chilvers and Corkeron 2002; Whitehead 2008b), we only considered those encountered on ≥ 4 occasions *and* in ≥ 2 different years. This restricted the analysis to animals with some meaningful level of site fidelity and removed transient individuals, to ensure an accurate representation of the social network. Although most authors limit analyses to animals with some arbitrary number of *total* sightings (Quintana-Rizzo and Wells 2001; Chilvers and Corkeron 2002; Pace et al. 2012), we further limited this to animals encountered in more than 1 year. This was because several animals seen multiple times were only seen in a single year and, therefore, considered visitors/transients. Our restriction criteria resulted in 38 individuals used in the analysis. This subset represents regular individuals (‘residents’) and is considered representative for this local population. Thirty-two animals (84.2%) were seen ≥ 5 times and 18 (47.4%) ≥ 10 times. Mean number of sightings per individual was 14 (SD 11.3, range 4–41). Multiple encounters during same day were only included if they were of different groups. Mother-dependent calves were excluded due to non-independence.

### Testing association patterns and network analysis

To minimise bias and facilitate comparisons, the half-weight association index (HWI) was used (Cairns and Schwager 1987). Although an attempt was made to photograph all members of each group, this was not always possible and the HWI accounts for this. It was recently suggested that a new index accounting for gregariousness (HWIG) may be more suitable (Godde et al. 2013). We also analysed associations using HWIG, but found little difference in results. Therefore, and to facilitate comparisons with previous studies, only HWI results are presented.

Analyses were performed in program SOCPROG 2.4 (Whitehead 2009). To test whether dyads (pairs of individuals) associated more often than expected by chance, we used the Manly-Bejder permutation technique (Manly 1995; Bejder et al. 1998) with extensions (Whitehead 1999; Whitehead et al. 2005) and corrections (Krause et al. 2009b). We used day as a sampling period. We generated 20,000 permutations (associations within samples) to ensure stability of *P* values. We also performed another round of permutations, with sampling period of 5 days, because permutations are often impossible (or perform poorly) with too few associations within a period.

We used standardised lagged association rates (SLAR, Fig. 3) to estimate the probability of dyads associated at a given time still being associated after a time lag and assess the stability of associations (Whitehead 1995). Precision (SE) was estimated by jack-knifing on each sampling period (Whitehead 2008a). To test for preferred/avoided associations, we compared SLAR to null association rate, which represents expected values for random associations (Whitehead 1995). A moving average enabled the optimal adjustment between precision and smoothing. Exponential models of social organisation developed by Whitehead (1995) were fitted to SLAR. Model selection was based on minimising the Quasi-Akaike Information Criterion (QAIC) (Burnham and Anderson 2002).

We calculated the social differentiation (*S*) to evaluate the level of variation in dyadic probability of association, i.e. how differentiated the network was (Whitehead 2008a). We calculated the correlation between true and estimated association indices (*r*) to evaluate if data accurately represented the true social network (Whitehead 2008b). To investigate existence of clusters and delineate units within the network, we carried out modularity analyses (Newman 2004) by applying the eigenvector method of Newman (2006), the knot-diagram analyses and the modularity-G (Whitehead 2008a). With this approach the animals were assigned to clusters so that the separation between clusters was maximised (Whitehead 2008a). To evaluate if association rates were similar within/between clusters, and within/between sexes, we compared mean association rates via two-tailed Mantel test.

To facilitate comparisons with other studies, we calculated several network metrics—HWI, Affinity, Betweenness, Closeness, Clustering coefficient, Eigenvector centrality, Reach and Strength (Wey et al. 2008; Whitehead 2008a)—for the entire network, individual clusters and for individuals (Table 2). These are measures of how well-connected and central individuals are (Whitehead 2008a).

**Table 2:** Social network metrics (mean ± SD) of individual social clusters

Metric	Cluster A (*n* = 19)	Cluster B (*n* = 13)	Cluster C (*n* = 6)	Overall (*n* = 38)
Mean HWI	0.21 ± 0.03	0.19 ± 0.03	0.06 ± 0.03	0.18 ± 0.06
Affinity	7.74 ± 0.14	7.04 ± 0.12	4.21 ± 0.86	6.94 ± 1.29
Betweenness	13.3 ± 16.6	5.04 ± 7.9	8.08 ± 8.46	9.68 ± 13.38
Closeness	52.37 ± 5.36	58.38 ± 4.93	64.67 ± 8.79	56.37 ± 7.28
Clustering coefficient	0.39 ± 0.06	0.45 ± 0.04	0.23 ± 0.03	0.39 ± 0.09
Eigenvector centrality	0.21 ± 0.04	0.09 ± 0.01	0.02 ± 0.01	0.14 ± 0.08
Reach	59.26 ± 9.69	48.82 ± 6.01	9.13 ± 5.25	47.77 ± 19.27
Strength	7.66 ± 1.27	6.95 ± 0.93	2.17 ± 0.96	6.55 ± 2.23

*HWI* half-weight association index, *n* number of animals

A social network diagram (Fig. 4) was created using NetDraw 2.123 (Borgatti 2002) to illustrate relationships and network structure. Nodes with highest associations are grouped together, while those with fewer links remain on the periphery. We created one diagram with all associations, regardless of strength (Fig. 4a), and one displaying only those with HWI greater than twice the overall mean (Fig. 4b), believed to represent meaningful associations (Durrell et al. 2004; Gero et al. 2005; Wiszniewski et al. 2012).

**Fig. 2:**
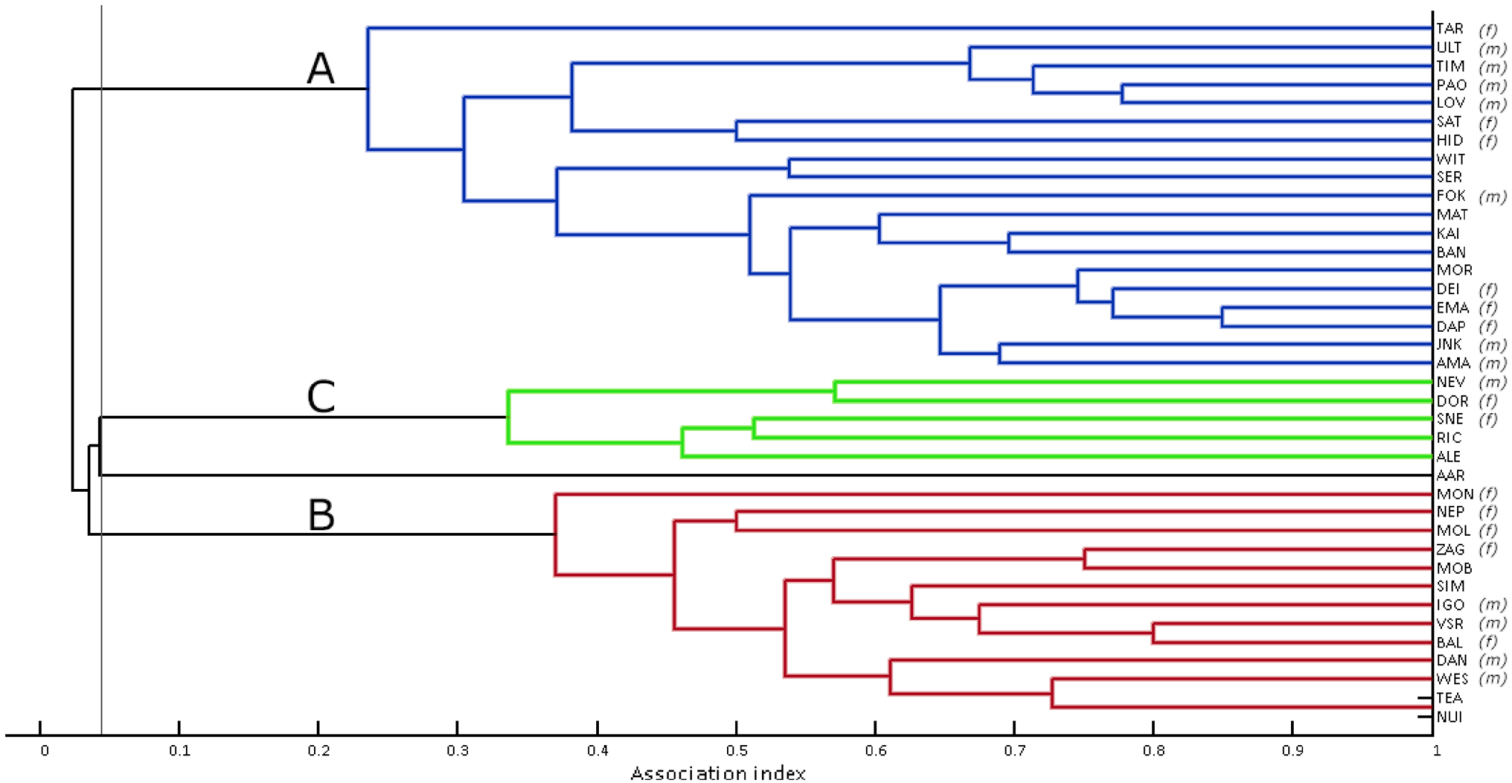
Dendrogram produced using average-linkage hierarchical cluster analysis (CCC = 0.96) for 38 common bottlenose dolphins. The clusters A, B and C represent clusters of animals based on modularity analyses with the eigenvector method of Newman (2006). The modularity-G of 0.464 suggests that the best division into clusters is with an association index of 0.043 (thin vertical line). Note that the dolphin AAR is not included in any cluster

Social structure was also represented with hierarchical average linkage cluster analysis (dendrogram, Fig. 2). Since dendrograms can be over-interpreted, especially if the society is not hierarchically arranged, we used strength of cophenetic correlation coefficient (CCC) to indicate whether the data interpretation was valid (Whitehead 2008a).

### Sex composition

To further examine potential sex segregation, we selected sightings involving at least two known-sex animals (including sightings with no or single known-sex individual would introduce a bias in estimating sex composition). In this sub-sample, we determined the proportion of male-only, female-only and mixed-sex groups.

## Results

### Association patterns

We photographed 132 dolphin groups, but the restriction criteria resulted in 115 encounters of 38 individuals included in analysis. All individuals were observed in the core study area, but could also be encountered elsewhere.

**Fig. 3:**
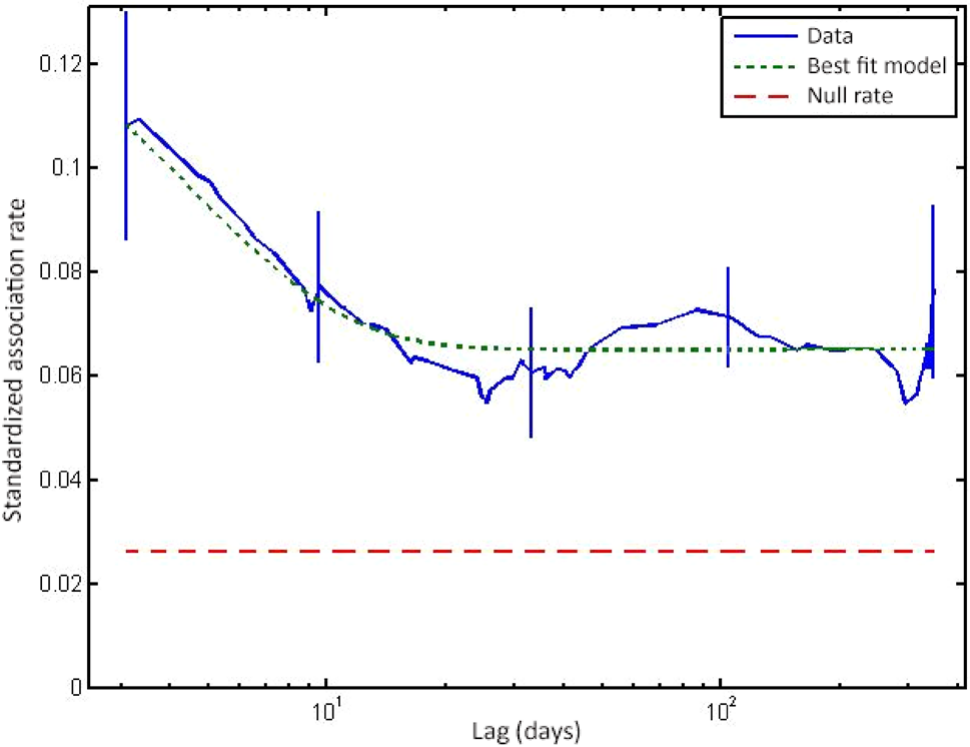
Standardized lagged association rate (SLAR) for 38 common bottlenose dolphins. A moving average of 8000 associations was used. Vertical bars indicate standard errors calculated using the temporal jackknife method on each sampling period. The best fit model (dotted line) indicates a social system model of ‘constant companions and casual acquaintances’. The null association rate (dashed line) represents the theoretical SLAR if individuals associated randomly

Network metrics are shown in Table 2. Correlation between true and estimated association indices (*r* ± SE = 0.840 ± 0.040, based on bootstrap with 10,000 replications), suggests that the data accurately describe the true social network (Whitehead 2008b). *P* values stabilised after about 9000 permutations. Standard deviation of the calculated (observed) associations was significantly higher than that of permuted data (observed SD = 0.236, random SD = 0.228, *P *< 0.001), as was the CV (observed CV = 1.259, random CV = 1.226, *P *< 0.001), indicating that associations were non-random (Gowans et al. 2001; Lusseau et al. 2003). Figure 2 shows that most dolphins had preferred associates, with one pair (NUI-TEA) always recorded together. CCC of 0.96 suggests a good fit and thus a good representation of true social structure (Whitehead 2008a). SLAR was best described by the so-called ‘constant companions and casual acquaintances’ model (Fig. 3, Table 3). SLAR line never reached the null association rate, indicating the absence of random associations and a high probability of dyads associated even after a prolonged time lag.

**Table 3:** Fit of social system models to the standardised lagged association rate (SLAR)

Model	Formula	Number of parameters	QAIC	ΔQAIC
CC	0.068	1	48,999.91	83.13
CA	0.069552e^−0.00002585*τ*^	2	48,999.97	83.19
CC + CA	0.066285 + 0.091054e^−0.25144*τ*^	3	48,916.78	0
Two levels of CA	0.24804e^−0.85368*τ*^ + 0.066852e^0.000003792*τ*^	4	48,943.40	26.63

*τ* represents time lag in days. The lowest Quasi-Akaike Information Criterion (QAIC) indicates the best-fitting model, and ΔQAIC (difference between QAIC and that of the best model) indicates the degree of support for the other models

*CC* constant companions, *CA* casual acquaintances

### Division of social network

**Fig. 4:**
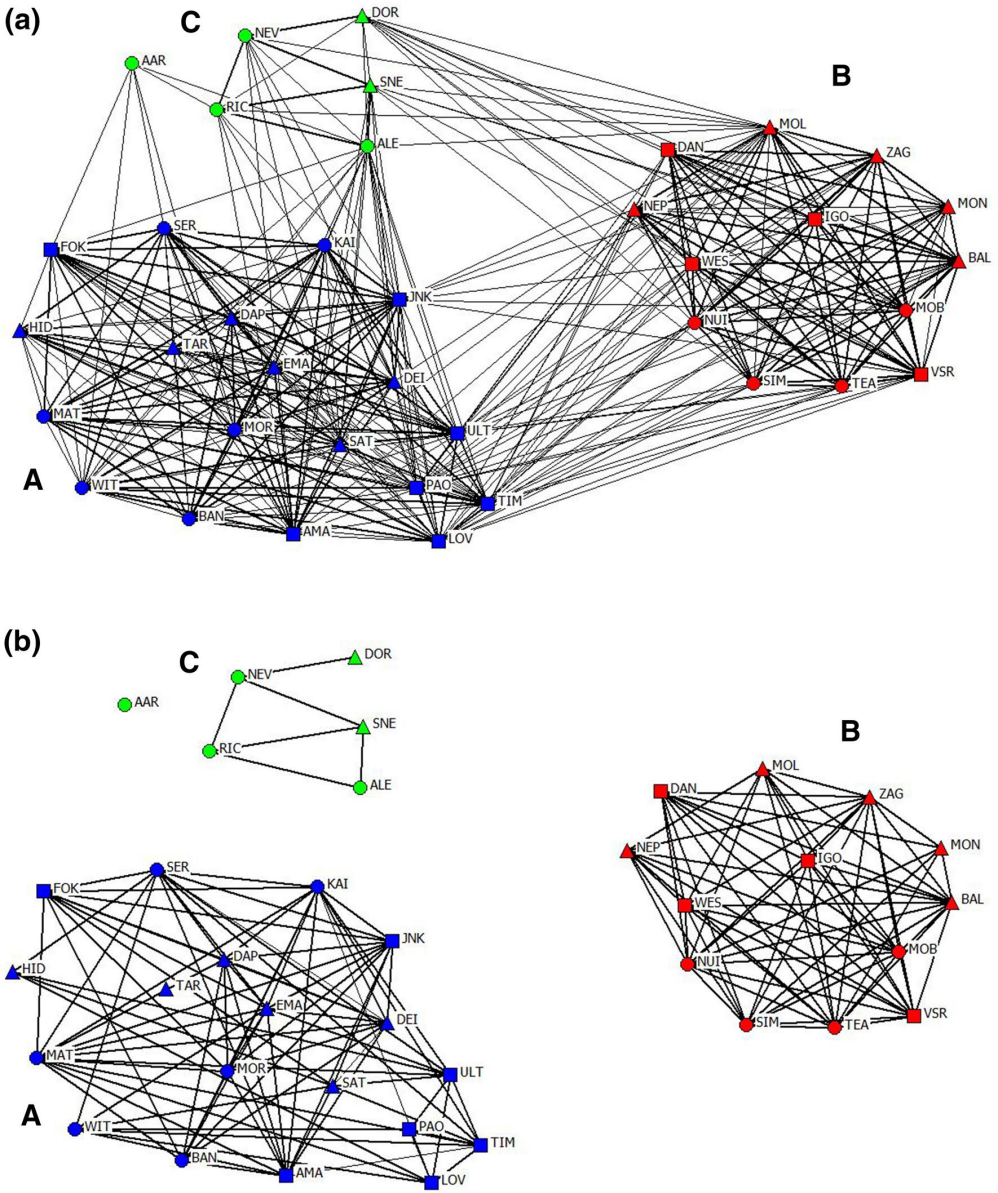
Social network diagram of the common bottlenose dolphin population. Nodes represent individuals (filled square = males, filled triangle = females, filled circle = unknown sex). Lines between nodes represent associations between dyads and the thickness of lines indicates the strength of relationship (value of an association index between dyads). Division of clusters is based on eigenvector method of Newman (2006) and modularity from gregariousness analyses. Cluster A = blue nodes, cluster B = red nodes, cluster C = green nodes. Note that individual AAR is included in cluster C but does not fit into it well. **a** All recorded associations between dyads, regardless of strength. **b** Only associations higher than twice the mean HWI (see main text for details)

Although the network was fluid overall, social differentiation estimate using likelihood method (*S* 1.076, SE 0.025) indicates a well-differentiated society (Whitehead 2008a). Average linkage cluster analysis (Fig. 2) and network analysis (Fig. 4) both showed a clear division into three distinct clusters, with one individual (AAR) not fully belonging in any. Modularity analysis assigned individuals to clusters with significantly higher associations within than between clusters (two-tailed Mantel test: *t* = 21.25, *P* = 1.0). Modularity-G division (peak at 0.464) suggests that the best division is with an association index of 0.043. The modularity-G peak suggests that with this division, there is much more total association within clusters than would be expected for randomly determined clusters. Since modularity values > 0.3 suggest a meaningful division (Newman 2004), the value of 0.464 provides compelling evidence of a structured network.

Dolphins formed two main clusters, A (19 individuals) and B (13 individuals), with strong associations, and a smaller cluster C (6 individuals) with much weaker associations (Table 2). Mean HWIs were similar between A and B and lower in cluster C (Table 2). Dolphins were predominantly found with other members of the same cluster, although group sizes varied. Cluster A and B dolphins were usually seen in large groups (> 10 and up to 45 individuals). Cluster A dolphins rarely interacted with those from cluster B (4/115 encounters, or 3.5%). These interactions never involved the majority of both clusters. Instead, while one (either A or B) featured the majority of animals, the other was represented by few (1–4).

Cluster C contained individuals that occasionally interacted with clusters A and B, but were typically seen with other cluster C animals, on their own, or with transient dolphins. They were predominantly found in small groups (2–3) or alone. They had no particularly strong bonds with anyone. Individual AAR did not fit into any cluster well (Fig. 2), but was placed in cluster C based on modularity, extremely low mean HWI of 0.01, an eigenvector value close to zero, and other network metrics.

Of 115 encounters, 55 (47.8%) included only cluster A animals, 10 (8.7%) included only cluster B animals and 37 (32.2%) included only cluster C animals. Three encounters (2.6%) included a mix of clusters A and B, 6 (5.2%) of clusters A and C, 3 (2.6%) of clusters B and C and 1 (0.9%) of all three clusters.

### Network metrics

Affinity, Clustering coefficient, Eigenvector centrality, Reach and Strength were comparable between clusters A and B and lower in cluster C. Individuals in A and B had more associates and formed more stable associations with them than those in C. Conversely, cluster C had a higher Closeness, which is a different measure of centrality—as cluster C animals interacted with both A and B, their shortest paths to all other individuals were shorter than for other two clusters. Finally, cluster B had the lowest Betweenness centrality, which is likely a combination of cluster size and how often its members interacted with other clusters.

### Sex segregation and sex differences

Twenty-five animals were sexed (13 females, 12 males). We found no evidence of sex segregation. Cluster A contained 6 females, 7 males, and 6 unknown sex animals; cluster B contained 5 females, 4 males, and 4 unknown sex animals; and cluster C contained 2 females and 4 unknown sex animals (Fig. 4).

Among groups where sex of at least two animals was known (*n* = 60), 76.7% were mixed-sex. This is likely an underestimate, as groups classified as ‘single-sex’, but involving unsexed individuals, could in fact be mixed. Among groups composed only of cluster A dolphins (*n* = 44), 81.8% were mixed-sex. We could not estimate this for clusters B and C, as the number of encounters with at least two sexed animals was insufficient.

Mean HWI was higher for male–male pairs (HWI ± SD = 0.25 ± 0.07) than male–female pairs (0.21 ± 0.05) and female–female pairs (0.15 ± 0.06), but differences between sexes were not significant (two-tailed Mantel test: *t* = − 0.916, *P* = 0.16).

### Temporal habitat use patterns

**Fig. 5:**
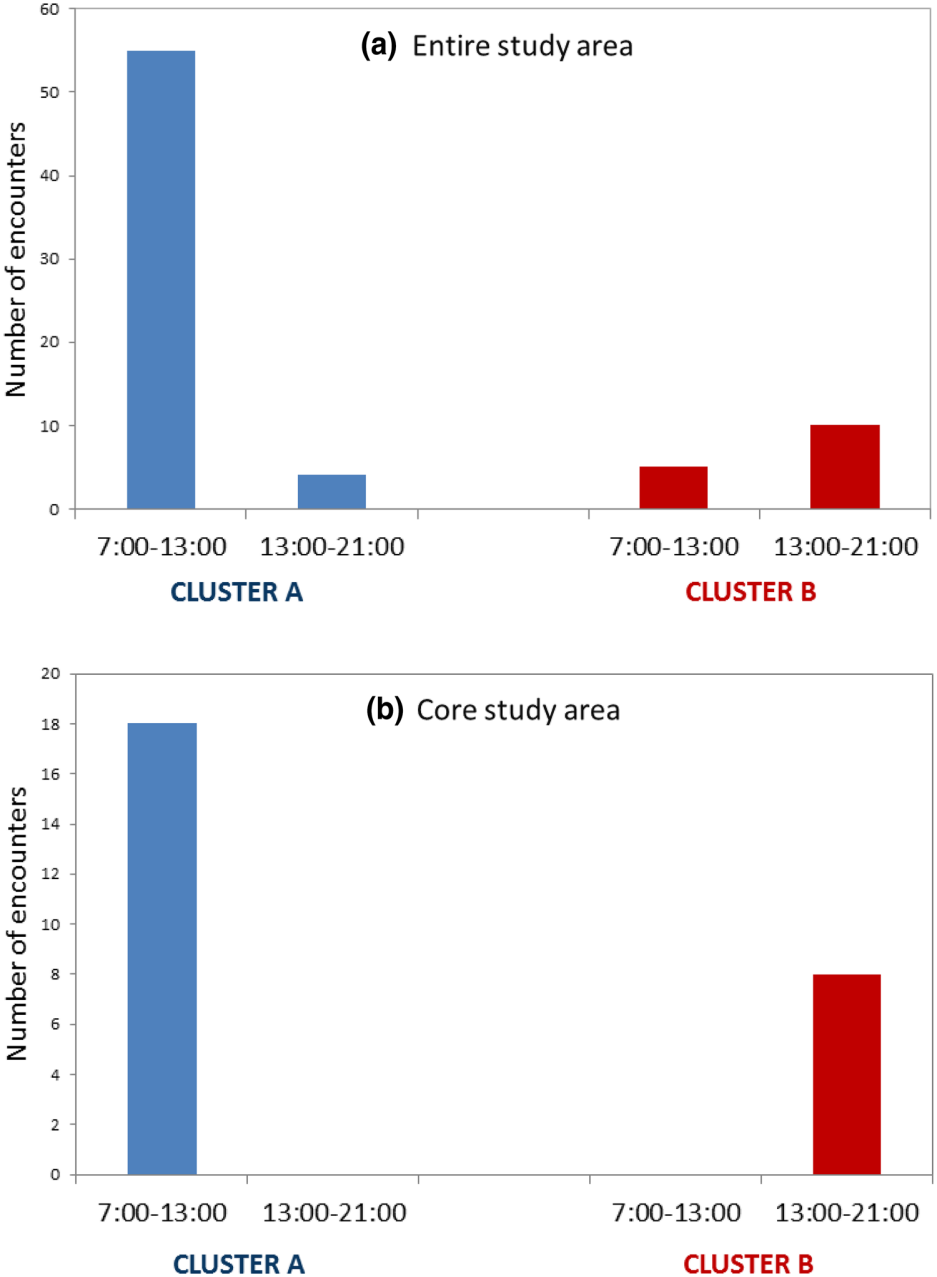
Temporal occurrence of clusters A and B in the **a** entire study area and **b** core study area

When we examined temporal (diel) occurrence patterns, an interesting trend became apparent. Clusters A and B overlapped spatially, but not temporally. They were almost never seen together, apart from four encounters mentioned earlier. Furthermore, while both regularly used the core study area, they used it at different times of day: cluster A was predominantly sighted in morning hours (07:00–13:00) and cluster B only in late afternoon hours (18:00–21:00). This trend was consistent in the core study area without deviation, although cluster A could be found elsewhere in the afternoon and cluster B could be found elsewhere in the morning. To test if there was any real pattern, we first looked at hours of occurrence of the two clusters for the entire study area. Next, to avoid bias resulting from different spatial preferences of dolphins or the spatial coverage of our survey effort, we looked at hours of occurrence in the core study area only (i.e. the area regularly covered by both boat-based and land-based surveys). For those few occasions when animals from more than one cluster were together, we assigned a group to a given cluster if it was predominantly composed of that cluster. When considering the entire study area and groups composed only or predominantly of cluster A dolphins, 55 encounters (93.2%) were between 07:00 and 13:00, and only 4 encounters (6.8%) after 13:00 (*n* = 59). Looking at the core study area only, all encounters (100%) of cluster A groups were before 13:00 (*n* = 18, Fig. 5). When considering the entire study area and groups composed only or predominantly of cluster B dolphins, 5 encounters (33.3%) occurred before 13:00, while the remaining 10 (66.7%) occurred after 18:00 (*n* = 15). Looking at the core study area only, all encounters (100%) of cluster B groups were recorded after 18:00 (*n* = 8; Fig. 5). The temporal use of the entire study area differed significantly between clusters A and B, as did the use of the core study area (Fisher’s exact test: *P* < 0.001).

Cluster C groups did not display such patterns. In the entire study area, 23 (62.2%) encounters of cluster C groups were before 13:00, while 14 (37.8%) were after 13:00 (*n* = 37). In the core study area, 20 (66.7%) were before 13:00, while 10 (33.3%) were recorded after (*n* = 30).

### Interactions with trawlers

Forty-eight interactions with trawlers were recorded during the study, of which 35 were during dolphin encounters considered in the analysis. Encounters involving trawler interactions accounted for 29.6% of dolphin encounters. Twenty-two (62.9%) of these interactions were with pelagic pair trawlers and 13 (37.1%) with bottom trawlers (one encounter involved interactions with both).

**Fig. 6:**
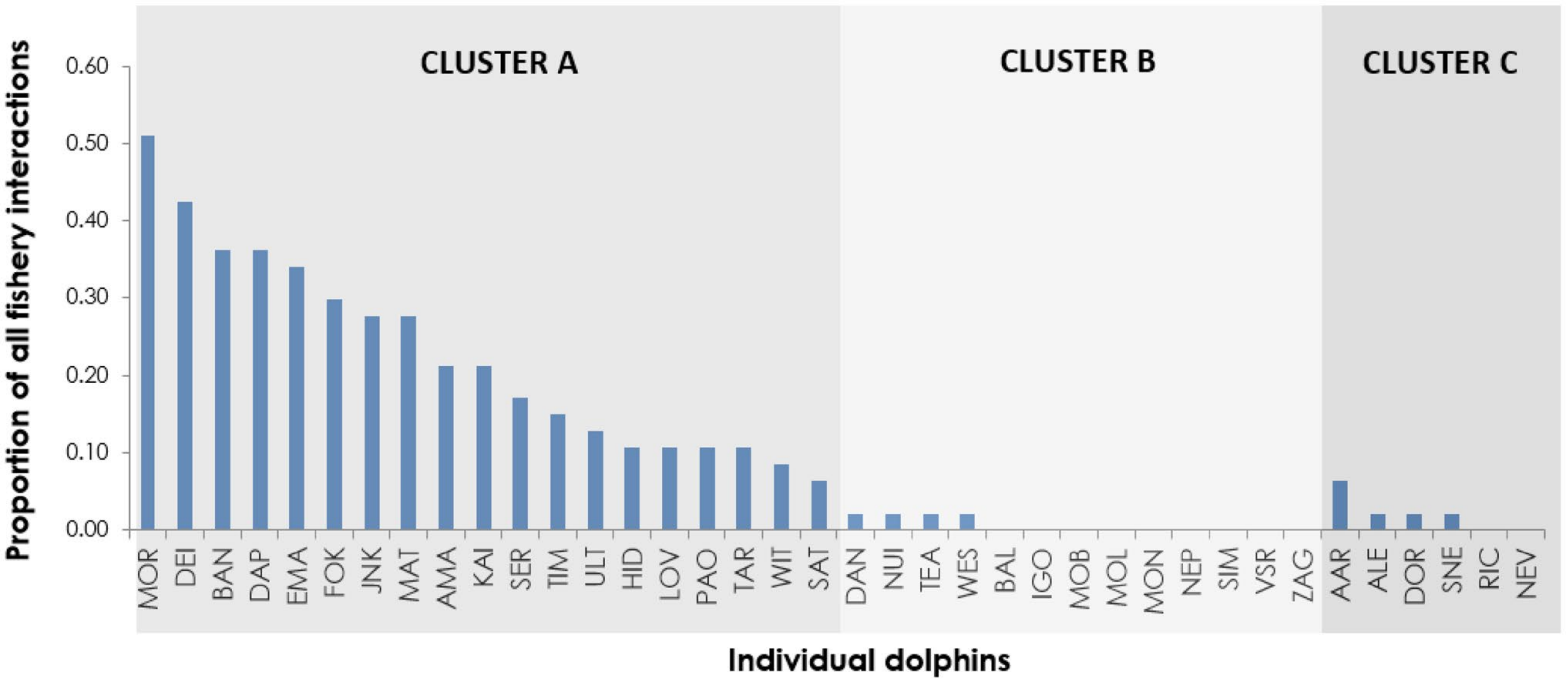
Proportion of all dolphin–fishery interactions an individual dolphin was recorded in

Majority of interactions with trawlers involved cluster A dolphins, with one individual (MOR) present in more than 50% of all interactions (Fig. 6). Mean number of interactions with any trawlers per individual in cluster A was 10.6 (SD 6.1, range 3–24). Twenty-eight interactions involved only cluster A dolphins (82.4%), 4 involved cluster A and cluster C dolphins (11.8%), 1 involved only cluster C dolphins (2.9%) and 1 involved cluster B and cluster C dolphins (2.9%). No interactions involved only cluster B dolphins.

Cluster B dolphins were never observed interacting with pair trawlers, while four individuals apparently interacted with a bottom trawler on one occasion. Mean number of interactions with any trawler per individual in this cluster was 0.31 (SD 0.48, range 0–1).

Dolphins from cluster C interacted with trawlers at intermediate level. Only one animal from cluster C (ALE) ever interacted with pair trawlers. This happened on one occasion, when the individual was with cluster A dolphins. On another occasion, the same individual was observed diving (*sensu* Bearzi et al. 1999) with another unidentifiable adult, when active pair trawlers passed by. The animals appeared to ignore them and continued diving in the same location. Other animals from cluster C were either never observed interacting with trawlers, or only observed interacting with bottom trawlers (Fig. 6). Mean number of interactions with any trawlers per individual in this cluster was 1 (SD 0.01, range 0–3).

## Discussion

### General social structure

Gulf of Trieste dolphins appear to live in two general kinds of social units: (a) large mixed-sex groups with strong, long-lasting associations and (b) small groups with weaker, temporally unstable associations. This does not appear to be age-dependent. Two largest clusters featured strong bonds, while seldom interacting with the other cluster. This structuring was also evident in the field. These two clusters showed high levels of group stability, which persisted through the study years and beyond (T. Genov, *personal observation*), although exact group membership could vary. Gregariousness, connectedness and strength of associations (indicated by HWI, Affinity, Clustering coefficient, Eigenvector centrality and Strength) were quite high and relatively similar between the two, as was the number of associates (Reach; Table 2). In contrast, these metrics were substantially lower in cluster C, where animals showed no strong association preferences. Because they were occasionally observed with animals from other clusters, their Closeness was highest (Table 2).

When including all associations (Fig. 4a) the network was reasonably well-connected, with no individual ‘bottlenecks’ between clusters, which were inter-connected via several but not particularly numerous individuals. Such ‘social brokers’ (Lusseau and Newman 2004) may maintain population cohesiveness and prevent complete cluster isolation, possibly having disproportionate influence on the population connectedness, as found in killer whales (Williams and Lusseau 2006), macaques (Flack et al. 2005) and squirrels (Manno 2008). However, when considering only ‘meaningful’ associations, greater than twice the mean HWI (Durrell et al. 2004; Gero et al. 2005; Wiszniewski et al. 2012), structuring becomes striking and clusters completely separated (Fig. 4b).

Associations were temporally relatively stable (as supported by SLAR and field observations), although stability varied with different levels of social organisation. Cluster A in particular (but also B) seemed to contain ‘core’ membership (first-level unit) and other ‘tiers’ that joined core members to form higher-level units. In such multi-level systems, seen also in African elephants (Wittemyer et al. 2005) clusters can sub-split during times of ecological constraints and fuse again when conditions are favourable or promote cooperation. We sometimes observed cluster A dolphins forming smaller groups (≤ 10), which often joined into groups of 30 + animals. Group composition during encounters was also surprisingly stable, more than in the closest other known local population in the Adriatic Sea (Bearzi et al. 1997) or in most other populations worldwide (Connor et al. 2000; Lusseau et al. 2006). Once encountered, groups were unlikely to change during observations, which could last several hours (Genov et al. 2008). This population is rather small (Genov et al. 2008; Genov 2011) and some authors hypothesised that community size influences group stability in fission–fusion societies, with smaller communities leading to decreased fission–fusion flexibility (Lehmann and Boesch 2004; Augusto et al. 2012).

In several *Tursiops* populations, social structure involves sex/age segregation (Wells et al. 1987; Connor et al. 2000; Fury et al. 2013). Here, structuring did not appear sex-related, as clusters contained both sexes. We found no evidence of male alliances. Although male–male associations were stronger than male–female or female–female associations, this was not significant, with stronger male–female than female–female associations. Most encountered groups contained both sexes (regardless of season), which suggests that mixed-sex groups were not related to reproductive state. Likewise, although more than half of all groups contained calves, adult-only groups were common. Reproductive state or presence of calves, therefore, fails to explain these patterns.

Presence of large mixed-sex groups resembles Doubtful Sound bottlenose dolphins in New Zealand (Lusseau et al. 2003). Lusseau et al. (2003) hypothesised that ecological constraints, such as variable productivity, drive social organisation. In such environments, groups may need to rely on individuals with long-term knowledge about spatio-temporal distribution of prey sources, which might explain lack of sex segregation and greater population connectedness (Lusseau et al. 2003). The northern Adriatic is characterised by large spatio-temporal variability in nutrient input and productivity (Fonda Umani et al. 2005; Mozetič et al. 2010, 2012), and our study area contains relatively uniform bottom topography. With lack of major prey-aggregating bottom features, spatio-temporal distribution of prey is likely highly variable, which may promote network connectedness. Clusters A and B both contained individuals which appeared ‘older’ based on their external appearance. These animals may possess long-term knowledge needed to tackle such constraints and thus play a key role in their community.

### Temporal segregation

Several studies found spatial segregation in *Tursiops* (Chilvers and Corkeron 2001; Chilvers et al. 2003; Lusseau et al. 2006; Fury et al. 2013; Carnabuci et al. 2016). In Moray Firth, Scotland, this segregation appeared season-dependent (Wilson et al. 1997). During summer, part of the population moved into inner parts of the Firth and was replaced by dolphins from outer parts. However, clusters in our study overlapped spatially, but not temporally, and we found differences on a daily, rather than seasonal level. Such intraspecific diel temporal partitioning does not appear to have been documented in cetaceans previously, nor in other mammals (Kronfeld-Schor and Dayan 2003), with one exception recorded in the use of running wheel in captive mice (Howerton and Mench 2014). Whether this pattern results from competitive exclusion, avoidance of aggressive interactions, or different foraging tactics, remains unknown. Given that prey resources in the marine environment are patchy and variable, prey resource defence is not a likely explanation (Ramp et al. 2010). Lack of sex segregation also dismisses access to females as an explanation. We are currently working to determine if genetic relatedness correlates with the social partitioning observed here.

We considered potential confounding factors. If the distribution of cluster A was linked to trawlers, which only operated during certain hours, this would explain the pattern. However, pair trawlers operated in the morning and afternoon, and bottom trawlers operated day-long (including evenings). Cluster A regularly used trawling areas even in the absence of trawlers, with no difference in group composition. More importantly, no trawlers operated in the core study area. Finally, cluster A dolphins did not *always* follow trawlers, even if trawlers were around. Trawlers, therefore, fail to explain temporal partitioning.

We also considered lower sample size for cluster B. Caution is needed when making inferences from small sample sizes, but temporal patterns here appear quite striking. The presence of a temporal (rather than spatial) pattern suggests the observed associations were not an artefact of space use (animals being together just because they use the same space), but due to genuine social preferences. Further, due to long-term and extensive survey effort (Table 1, Fig. 1), this pattern is unlikely to be an artefact of effort. Surveys in recent years (2012–2017, analysis pending) further support this, with both clusters continuing this pattern, and even occurring in the same area within a single day, but at different times (Morigenos, unpublished data).

Finally, it remains to be determined if segregation is specific to this area, or if it occurs in other areas used by the animals. The range of this local population is unknown (Genov et al. 2016), but evidence from photo-ID (Genov et al. 2009) and genetics (Gaspari et al. 2015) suggest it is a distinct unit.

### Interactions with trawlers

Two clusters displayed behavioural differences related to trawling. Cluster A dolphins often interacted with pair trawlers and occasionally bottom trawlers, while cluster B dolphins did not (‘trawler dolphins’ vs. ‘non-trawler dolphins’, Chilvers and Corkeron 2001). Fishing has a major impact on cetaceans worldwide, not only through incidental mortality (Read et al. 2006), but also through prey depletion (Bearzi et al. 2008), habitat degradation (Turner et al. 1999) and ecosystem change (Worm et al. 2006). More subtly, fishing activities can affect, or be affected by, cetacean behaviour. In Queensland, Australia, bottlenose dolphins were found to form two communities, where one fed in association with trawlers and the other did not (Chilvers and Corkeron 2001; Chilvers et al. 2003). Following fishery closure, dolphins restructured and homogenised their network, suggesting that structuring was fishery-induced (Ansmann et al. 2012). Our study shows similarities, but also important differences. First, in the population studied by Ansmann et al. (2012), dolphins fed on discards, while our dolphins followed operating trawlers, presumably feeding actively inside/behind the net (Genov et al. 2008; Kotnjek 2016). Second, structuring in our study related to temporal rather than spatial segregation, and did not appear only fishery-related. Another study in the Mediterranean Sea related dolphin association patterns to bottom trawling and fish farming, but animals mixed more frequently than ours (Pace et al. 2012).

Human activities can likely alter behaviour and social structure of mammals (Rutledge et al. 2010; Ansmann et al. 2012) and this may well be the case here. However, causal links are unclear and it is difficult to ascertain what came first. The inherent social structure itself, and social learning, may lead to differential behaviour and interactions with anthropogenic activities, without these activities changing the social system in the first place. It is interesting to note that the pair trawler fishery in our area closed in 2012. This did not appear to change associations or temporal patterns, but cluster A did appear to increase rates of interactions with bottom trawlers (Morigenos, unpublished data).

Diet information for this population is limited, but dietary preferences may explain different fishery-related foraging tactics. Both clusters were observed taking mullets (*Mugil*/*Liza* sp., Genov et al. 2008, Morigenos, unpublished data) and both regularly feed in the core study area. Their diets, therefore, overlap, but to an unknown extent. However, the apparent ‘switch’ of cluster A to bottom trawlers after the closure of pair trawler fishery suggests that behavioural specialisation and hunting techniques, rather than prey preference, may be more likely. Our further research aims to provide better insight into the feeding ecology of this population through stable isotope analysis.

Whether interactions with trawlers increase fitness (by maximising energetic intake and minimising expenditure) or decrease it (through increased bycatch), is unknown. Both clusters produce new offspring and appear stable, and there is no evidence of trawler-related bycatch in this area.

## Conclusions

We show that local dolphins (1) form distinct social clusters; (2) exhibit temporal partitioning; and (3) differ in interactions with fisheries. We demonstrate how different segments of the same population may behave very differently and have differing effects on human activities such as fishing (through potential depredation or gear damage). In turn, they may respond differently to anthropogenic pressures, as temporal partitioning may make animals either more or less vulnerable to disturbance from boat traffic.

## References

[CR1] Ansmann IC, Parra GJ, Chilvers BL, Lanyon JM (2012). Dolphins restructure social system after reduction of commercial fisheries. Anim Behav.

[CR2] Augusto JF, Rachinas-Lopes P, dos Santos ME (2012). Social structure of the declining resident community of common bottlenose dolphins in the Sado Estuary. Port J Mar Biol Assoc UK.

[CR78] Bearzi G, Notarbartolo di Sciara G, Politi E (1997). Social ecology of bottlenose dolphins in the Kvarneric (northern Adriatic Sea). Mar Mammal Sci.

[CR3] Bearzi G, Politi E, Notarbartolo di Sciara G (1999). Diurnal behavior of free-ranging bottlenose dolphins in the Kvarnerić (northern Adriatic Sea). Mar Mamm Sci.

[CR4] Bearzi G (2008). Overfishing and the disappearance of short-beaked common dolphins from western Greece. Endanger Species Res.

[CR5] Bejder L, Fletcher D, Bräger S (1998). A method for testing association patterns of social animals. Anim Behav.

[CR6] Borgatti SP (2002). Netdraw network visualization.

[CR7] Burnham KP, Anderson DR (2002). Model selection and multimodel inference: a practical information-theoretic approach.

[CR8] Cairns SJ, Schwager SJ (1987). A comparison of association indices. Anim Behav.

[CR9] Cantor M, Whitehead H (2013). The interplay between social networks and culture: theoretically and among whales and dolphins. Philos Trans R Soc B Biol Sci.

[CR10] Carnabuci M, Schiavon G, Bellingeri M, Fossa F, Paoli C, Vassallo P, Gnone G (2016). Connectivity in the network macrostructure of *Tursiops truncatus* in the Pelagos Sanctuary (NW Mediterranean Sea): does landscape matter?. Popul Ecol.

[CR11] Chapman CA, Rothman JM (2009). Within-species differences in primate social structure: evolution of plasticity and phylogenetic constraints. Primates.

[CR12] Chilvers BL, Corkeron PJ (2001). Trawling and bottlenose dolphins’ social structure. Proc R Soc B Biol Sci.

[CR13] Chilvers BL, Corkeron PJ (2002). Association patterns of bottlenose dolphins (*Tursiops aduncus*) off Point Lookout, Queensland, Australia. Can J Zool.

[CR14] Chilvers BL, Corkeron PJ, Puotinen ML (2003). Influence of trawling on the behaviour and spatial distribution of Indo-Pacific bottlenose dolphins (*Tursiops aduncus*) in Moreton Bay, Australia. Can J Zool.

[CR15] Connor RC, Heithaus MR, Barre LM (1999). Superalliance of bottlenose dolphins. Nature.

[CR16] Connor RC, Wells RS, Mann J, Read AJ, Mann J, Connor RC, Tyack PL, Whitehead H (2000). The bottlenose dolphin: social relationships in a fission–fusion society. Cetacean societies: field studies of dolphins and whales.

[CR17] Connor R, Smolker R, Bejder L (2006). Synchrony, social behaviour and alliance affiliation in Indian Ocean bottlenose dolphins, *Tursiops aduncus*. Anim Behav.

[CR18] Connor RC, Watson-Capps JJ, Sherwin WB, Krützen M (2011). A new level of complexity in the male alliance networks of Indian Ocean bottlenose dolphins (*Tursiops* sp.). Biol Lett.

[CR19] Durrell JL, Sneddon IA, O’Connell NE, Whitehead H (2004). Do pigs form preferential associations?. Appl Anim Behav Sci.

[CR20] Flack JC, Krakauer DC, de Waal FB (2005). Robustness mechanisms in primate societies: a perturbation study. Proc R Soc B Biol Sci.

[CR21] Fonda Umani S (2005). Inter-annual variations of planktonic food webs in the northern Adriatic Sea. Sci Total Environ.

[CR22] Fury CA, Ruckstuhl KE, Harrison PL (2013). Spatial and social sexual segregation patterns in Indo-Pacific bottlenose dolphins (*Tursiops aduncus*). PLoS One.

[CR23] Gaspari S (2015). Drivers of population structure of the bottlenose dolphin (*Tursiops truncatus*) in the Eastern Mediterranean Sea. Evol Biol.

[CR24] Genov T (2011) Ecology of the bottlenose dolphin (*Tursiops truncatus*) in the northern Adriatic. Graduation thesis, University of Ljubljana

[CR25] Genov T, Kotnjek P, Lesjak J, Hace A, Fortuna CM (2008). Bottlenose dolphins (*Tursiops truncatus*) in Slovenian and adjacent waters (northern Adriatic Sea). Annales Series Historia Naturalis.

[CR26] Genov T, Wiemann A, Fortuna CM (2009). Towards identification of the bottlenose dolphin (*Tursiops truncatus*) population structure in the north-eastern Adriatic Sea: preliminary results. Varst Narave.

[CR27] Genov T (2016). Mid-distance re-sighting of a common bottlenose dolphin in the northern Adriatic Sea: insight into regional movement patterns. J Mar Biol Assoc UK.

[CR28] Genov T, Centrih T, Wright AJ, Wu G-M (2017). Novel method for identifying individual cetaceans using facial features and symmetry: a test case using dolphins. Mar Mamm Sci.

[CR29] Gero S, Bejder L, Whitehead H, Mann J, Connor R (2005). Behaviourally specific preferred associations in bottlenose dolphins, *Tursiops* spp. Can J Zool.

[CR30] Godde S, Humbert L, Côté SD, Réale D, Whitehead H (2013). Correcting for the impact of gregariousness in social network analyses. Anim Behav.

[CR31] Gowans S, Whitehead H, Hooker SK (2001). Social organization in northern bottlenose whales, *Hyperoodon ampullatus*: not driven by deep-water foraging?. Anim Behav.

[CR32] Heyes CM (1994). Social learning in animals: categories and mechanisms. Biol Rev.

[CR33] Howerton CL, Mench JA (2014). Running around the clock: competition, aggression and temporal partitioning of running wheel use in male mice. Anim Behav.

[CR34] Kotnjek P (2016) Interactions between bottlenose dolphins (*Tursiops truncatus*) and fishing activities in the northern Adriatic Sea. Graduation thesis, University of Ljubljana

[CR35] Krause J, Lusseau D, James R (2009). Animal social networks: an introduction. Behav Ecol Sociobiol.

[CR36] Krause S, Mattner L, James R, Guttridge T, Corcoran MJ, Gruber SH, Krause J (2009). Social network analysis and valid Markov chain Monte Carlo tests of null models. Behav Ecol Sociobiol.

[CR37] Kronfeld-Schor N, Dayan T (2003). Partitioning of time as an ecological resource. Ann Rev Ecol Evol Syst.

[CR38] Laland KN (2004). Social learning strategies. Anim Learn Behav.

[CR39] Lehmann J, Boesch C (2004). To fission or to fusion: effects of community size on wild chimpanzee (*Pan troglodytes verus*) social organisation. Behav Ecol Sociobiol.

[CR40] Lusseau D (2006). Evidence for social role in a dolphin social network. Evol Ecol.

[CR41] Lusseau D, Newman ME (2004). Identifying the role that animals play in their social networks. Proc R Soc B Biol Sci.

[CR42] Lusseau D, Schneider K, Boisseau OJ, Haase P, Slooten E, Dawson SM (2003). The bottlenose dolphin community of doubtful sound features a large proportion of long-lasting associations—can geographic isolation explain this unique trait?. Behav Ecol Sociobiol.

[CR43] Lusseau D (2006). Quantifying the influence of sociality on population structure in bottlenose dolphins. J Anim Ecol.

[CR44] Manly BF (1995). A note on the analysis of species co-occurrences. Ecology.

[CR45] Mann J, Sargeant B, Fragaszy DM, Perry S (2003). Like mother, like calf: The ontogeny of foraging traditions in wild Indian Ocean bottlenose dolphins (*Tursiops* sp.). The biology of traditions: models and evidence.

[CR46] Mann J, Connor RC, Tyack PL, Whitehead H (2000). Cetacean societies.

[CR47] Manno TG (2008). Social networking in the Columbian ground squirrel, *Spermophilus columbianus*. Anim Behav.

[CR48] Mozetič P (2010). Recent trends towards oligotrophication of the northern Adriatic: evidence from chlorophyll *a* time series. Estuaries Coasts.

[CR49] Mozetič P, Francé J, Kogovšek T, Talaber I, Malej A (2012). Plankton trends and community changes in a coastal sea (northern Adriatic): bottom-up vs. top-down control in relation to environmental drivers Estuarine. Coast Shelf Sci.

[CR50] Newman ME (2004). Analysis of weighted networks. Phys Rev E.

[CR51] Newman ME (2006). Modularity and community structure in networks. Proc Natl Acad Sci.

[CR52] Pace DS, Pulcini M, Triossi F (2012). Anthropogenic food patches and association patterns of *Tursiops truncatus* at Lampedusa island, Italy. Behav Ecol.

[CR53] Popa-Lisseanu AG, Bontadina F, Mora O, Ibáñez C (2008). Highly structured fission–fusion societies in an aerial-hawking, carnivorous bat. Anim Behav.

[CR54] Quintana-Rizzo E, Wells RS (2001). Resighting and association patterns of bottlenose dolphins (*Tursiops truncatus*) in the Cedar Keys, Florida: insights into social organization. Can J Zool.

[CR55] Ramp C, Hagen W, Palsbøll P, Bérubé M, Sears R (2010). Age-related multi-year associations in female humpback whales (*Megaptera novaeangliae*). Behav Ecol Sociobiol.

[CR56] Randić S, Connor RC, Sherwin WB, Krützen M (2012). A novel mammalian social structure in Indo-Pacific bottlenose dolphins (*Tursiops* sp.): complex male alliances in an open social network. Proc R Soc B Biol Sci.

[CR57] Read AJ, Drinker P, Northridge S (2006). Bycatch of marine mammals in US and global fisheries. Conserv Biol.

[CR58] Rutledge LY, Patterson BR, Mills KJ, Loveless KM, Murray DL, White BN (2010). Protection from harvesting restores the natural social structure of eastern wolf packs. Biol Conserv.

[CR59] Smith JE, Kolowski JM, Graham KE, Dawes SE, Holekamp KE (2008). Social and ecological determinants of fission–fusion dynamics in the spotted hyaena. Anim Behav.

[CR60] Smolker RA, Richards AF, Connor RC, Pepper JW (1992). Sex differences in patterns of association among Indian Ocean bottlenose dolphins. Behaviour.

[CR61] Turner SJ, Thrush S, Hewitt J, Cummings V, Funnell G (1999). Fishing impacts and the degradation or loss of habitat structure. Fish Manag Ecol.

[CR62] Wells RS, De Waal FBM, Tyack PL (2003). Dolphin social complexity: lessons from long-term study and life history. Animal social complexity: intelligence, culture, and individualized societies.

[CR63] Wells RS, Scott MD, Irvine AB, Genoways HH (1987). The social structure of free ranging bottlenose dolphins. Current mammalogy.

[CR64] Wey T, Blumstein DT, Shen W, Jordán F (2008). Social network analysis of animal behaviour: a promising tool for the study of sociality. Anim Behav.

[CR65] Whitehead H (1995). Investigating structure and temporal scale in social organizations using identified individuals. Behav Ecol.

[CR66] Whitehead H (1999). Testing association patterns of social animals. Anim Behav.

[CR67] Whitehead H (2008). Analyzing animal societies: quantitative methods for vertebrate social analysis.

[CR68] Whitehead H (2008). Precision and power in the analysis of social structure using associations. Anim Behav.

[CR69] Whitehead H (2009). SOCPROG programs: analysing animal social structures. Behav Ecol Sociobiol.

[CR70] Whitehead H (2010). Conserving and managing animals that learn socially and share cultures. Learn Behav.

[CR71] Whitehead H, Bejder L, Andrea Ottensmeyer C (2005). Testing association patterns: issues arising and extensions. Anim Behav.

[CR72] Williams R, Lusseau D (2006). A killer whale social network is vulnerable to targeted removals. Biol Let.

[CR73] Wilson B, Thompson PM, Hammond PS (1997). Habitat use by bottlenose dolphins: seasonal distribution and stratified movement patterns in the Moray Firth, Scotland. J Appl Ecol.

[CR74] Wiszniewski J, Brown C, Möller LM (2012). Complex patterns of male alliance formation in a dolphin social network. J Mammal.

[CR75] Wittemyer G, Douglas-Hamilton I, Getz WM (2005). The socioecology of elephants: analysis of the processes creating multitiered social structures. Anim Behav.

[CR76] Worm B (2006). Impacts of biodiversity loss on ocean ecosystem services. Science.

[CR77] Würsig B, Jefferson TA (1990) Methods of photo-identification for small cetaceans. In: Hammond PS, Mizroch SA, Donovan GP (eds) Individual recognition of cetaceans: use of photo-identification and other techniques to estimate population parameters. Report of the International Whaling Commission, Special Issue 12, Cambridge, UK, pp 43–52

